# Legacy health effects among never smokers exposed to occupational secondhand smoke

**DOI:** 10.1371/journal.pone.0215445

**Published:** 2019-04-18

**Authors:** Eileen McNeely, Irina Mordukhovich, Steven Staffa, Samuel Tideman, Brent Coull

**Affiliations:** 1 Department of Environmental Health, Harvard T.H. Chan School of Public Health, Boston, MA, United States of America; 2 Department of Biostatistics, Harvard T.H. Chan School of Public Health, Boston, MA, United States of America; Telethon Institute for Child Health Research, AUSTRALIA

## Abstract

**Objectives:**

Secondhand tobacco smoke (SHTS) is a tremendous public health hazard, leading to morbidity and premature mortality worldwide, with racial and ethnic minorities and those of lower socioeconomic status disproportionately affected. Flight attendants were historically exposed to high levels of SHTS in the aircraft cabin. The health effects of active smoking are known to persist for up to a lifetime, but the legacy effects of SHTS exposure have not been well characterized.

**Design:**

We aimed to evaluate the legacy health effects of occupational SHTS exposure among never smoking workers using the resources of the Harvard Flight Attendant Health Study, a large study of cabin crew health. We evaluated associations between SHTS exposure and a range of diagnoses using multivariate logistic regression to calculate odds ratios (ORs) and 95% confidence intervals (CIs), employing a case-control sampling method and applying the bootstrap method to increase accuracy and precision of results.

**Results:**

We found no evidence of positive associations between SHTS and any cancer, but observed associations between SHTS and cardiac outcomes, including myocardial infarction (OR = 140, 95% CI: 1·04, 3·27) and peripheral artery disease (OR = 1·27, 95% CI: 1·00, 1·97). We also found associations between SHTS exposure and repeated pneumonia (OR = 1·06, 95% CI: 1·02, 1·10).

**Conclusions:**

Our study reports associations between legacy SHTS exposure going back decades and severe cardiac and respiratory health outcomes. Given the high prevalence of ongoing and historical SHTS exposure, our findings, if confirmed, have important implications for smoking cessation efforts, health education, and clinical guidelines.

## Introduction

Secondhand tobacco smoke (SHTS) is a tremendous public health hazard, leading to morbidity and premature mortality worldwide. Health effects of SHTS include lung cancer, cardiovascular disease (CVD), and impaired respiratory health [1–3]. Flight attendants were historically exposed to high levels of SHTS due to the past ubiquity of in-flight smoking, recirculated air and poor ventilation in the cabin, and an environment that increases respiratory stress through reduced oxygenation, elevated carbon dioxide, and low humidity [2]. A 1986 report by the National Academy of Sciences found that full-time flight attendants were exposed to SHTS at levels equivalent to living with a pack-a-day smoker [4]. When in-flight smoking was permitted, typical levels of respirable suspended particles violated current federal fine particulate matter standards threefold and exceeded irritation thresholds by ten to a hundred times, and SHTS exposure among cabin crew was six-fold that of the average U.S. worker and fourteen-fold that of the average U.S. resident [2].

In-flight smoking continued unabated in the U.S. until national bans began to be instituted in 1988 after decades of advocacy by flight crew, health organizations, and concerned citizens [5]. Smoking bans were initially limited to two-hour flights. In 1990, bans extended to domestic flights of up to six hours. Smoking bans were first implemented on U.S.-based international flights in 1995 and were implemented on nearly all flights to and from the U.S. by 1998 [5].

The health effects of active and passive smoking can persist for months to a lifetime after cessation of exposure, depending on the health outcome and an individual’s unique characteristics [1,3]. We aimed to evaluate the legacy health effects of SHTS exposure among never smoking flight attendants, an understudied occupational cohort historically exposed to high levels of SHTS, using the resources of a large, ongoing study of cabin crew health [6]. Our research is relevant to the many populations exposed to SHTS, is one of few studies to evaluate the legacy effects of workplace SHTS exposure, and to our knowledge is the most comprehensive study on this topic to date. Results from our research may inform guidelines among people with SHTS exposure histories with regard to preventative health, screenings and health education. We hypothesized that we would observe continued health effects of SHTS exposure among crew working today.

## Materials and methods

### Study population

Participants were enrolled in the second wave of the Harvard Flight Attendant Health Study (FAHS), a study of cabin crew health established in 2007 with 4,011 participants [6]. In 2014–2015, we recruited new and returning participants through a hard copy survey mailed to the original participants and an online survey launched in December 2014 [7]. We also conducted in-person recruitment at five U.S. airport hubs between December 2014 and June 2015, where we distributed postcards with the survey URL and hardcopy surveys. Our recruitment campaign included announcements from local unions, a study website, and a social media presence. Participants could enter a lottery to win an iPad or Apple watch as an incentive to enroll in our study. We modeled our mixed methods recruitment approach after high-profile studies using easily accessible and adaptable online surveys formatted for smart phones and tablets [8].

While the first wave of the FAHS recruited flight attendants from employment rosters at two U.S. airlines, any current or former flight attendant was eligible to participate in the second wave of our study. We collected 1,642 surveys from returning participants, yielding a 40% response rate from the original cohort with valid addresses. The 2014–2015 cohort enrolled a total of 5,922 U.S. participants with information on age and gender, though our effective study population comprised 3,015 participants due to restriction to people who could have potentially worked prior to the implementation of smoking bans. Our study was approved by the Harvard T.H. Chan School of Public Health Institutional Review Board. All participants provided their written informed consent. Because of participant privacy concerns and Institutional Review Board specifications, we do not provide individual participant’s data along with this manuscript.

### Survey

The original 2007 survey was developed from numerous focus groups with flight attendants and included validated questions about self-reported health outcomes and symptomology, work exposures and experiences, personal characteristics, and lifestyle factors taken from established surveys such as the Job Content Questionnaire and the National Health and Nutrition Examination Survey [9,10]. Dates of reported health outcomes were not recorded. Participants were also asked to provide employment history, including airlines, primary hubs, and dates of employment and leave. We updated the survey instrument in 2014–2015 to account for participant feedback and to refine our research questions based on earlier findings. Survey questions are shown in the Supporting Information (S1 Text).

### Exposure definition and statistical analysis

We calculated descriptive statistics for participant characteristics, presented as means and standard deviations or frequencies and percentages. To better understand the structure of our dataset, we evaluated the distribution of the age of starting work as a flight attendant and created a scatterplot of years of tenure in relation to SHTS exposure duration. We restricted our primary analysis to lifetime non-smokers to avoid having current or past smokers in the unexposed group. We also conducted sensitivity analyses among all participants (smokers and non-smokers). Health outcomes were treated as negative unless participants answered affirmatively to questions about diagnoses (i.e. questions receiving no response were recorded as a “no”). We used Stata version 15.0 (StataCorp, College Station, Texas) and R 3.2.2 for data management and analysis.

We evaluated associations between SHTS exposure and a range of diagnoses using multivariate logistic regression to calculate odds ratios (ORs) and 95% confidence intervals (CIs) [11]. We used a case-control sampling method, matching each case to three to four controls. In the primary analysis restricted to never smokers, matching criteria were same three-year birth window (ex: 1960–1962), gender (male/female), and race (white/non-white). In sensitivity analyses among the full cohort, additional matching criteria were current and past smoking (yes/no). We employed a complete case analysis and only included health outcomes that had a minimum of twenty diagnosed cases.

We restricted our dataset to currently employed cabin crew born before 1971 to avoid biased results from retired flight attendants having varying amounts of recovery time and to ensure that all participants could have started their careers before 1988 and hence have a possibility of high-level SHTS exposure. To further isolate the effect of SHTS, we adjusted for years of tenure as cabin crew minus leave (net tenure) and body mass index (BMI, in units of kg/m^2^) in all models.

We also conducted sensitivity analyses defining SHTS exposure according to years worked prior to 1990 and 1995, in order to take into account the gradual institution of smoking bans [5].

We used the bootstrap method for calculating ORs and CIs from multivariate analyses [12,13]. For each outcome, we ran 1,000 simulations resampling cases with replacement and reselecting the matching controls. The resulting point estimate is the median estimate from 1,000 simulations with a CI bounded by the 25^th^ percentile lowest and 75^th^ percentile highest estimate. We made this decision to reduce the variability resulting from random selection of matching controls. The bootstrap method yields more accurate point estimates and slightly more precise confidence intervals. The final sample for examining associations between health outcomes and one year of working in SHTS conditions was 3,015 among never smokers and 4,648 among all participants.

## Results

We present participant characteristics in Table 1, overall and stratified by SHTS exposure status. Never-smoking participants presented with a mean age of 56 years, mean job tenure of 24 years, and mean BMI of 24 kg/m^2^, and most were female (83%) and white (86%). While we did not conduct formal tests for heterogeneity by exposure status, most characteristics were similar between the groups, except for age (61 vs 53 years) and tenure (34 vs 17 years). Characteristics of combined ever and never smokers were similar to that of the restricted sample (S1 Table). Only seven percent of our full sample were current smokers, while 34% were past smokers.

**Table 1:** Demographics and baseline characteristics of lifetime non-smoking Harvard Flight Attendant Health Study participants born before 1971 (Wave 2: 2014–2015).

	Full Sample (n = 3,015)		Exposed (n = 1,294)		Unexposed (n = 1,721)	
Characteristic	N or Mean	% or SD	N or Mean	% or SD	N or Mean	% or SD
Age (years)	56·3	7·4	60·8	6·3	52·8	6·1
Net Tenure (years)	24·4	10·9	34·0	7·4	17·2	6·8
Net Exposure (years)	3·9	6·2	9·1	6·4	0	0
BMI (kg/m^2^)	24·3	4·1	24·2	4·2	24·3	3·9
Sex						
Male	511	17·0	179	13·8	332	19·3
Female	2,504	83·1	1,115	86·2	1,389	80·7
Race						
White	2,603	86·3	1,162	89·8	1,441	83·7
Non-White	412	13·7	132	10·2	280	16·3

BMI: body mass index; SD: standard deviation

**Fig 1:**
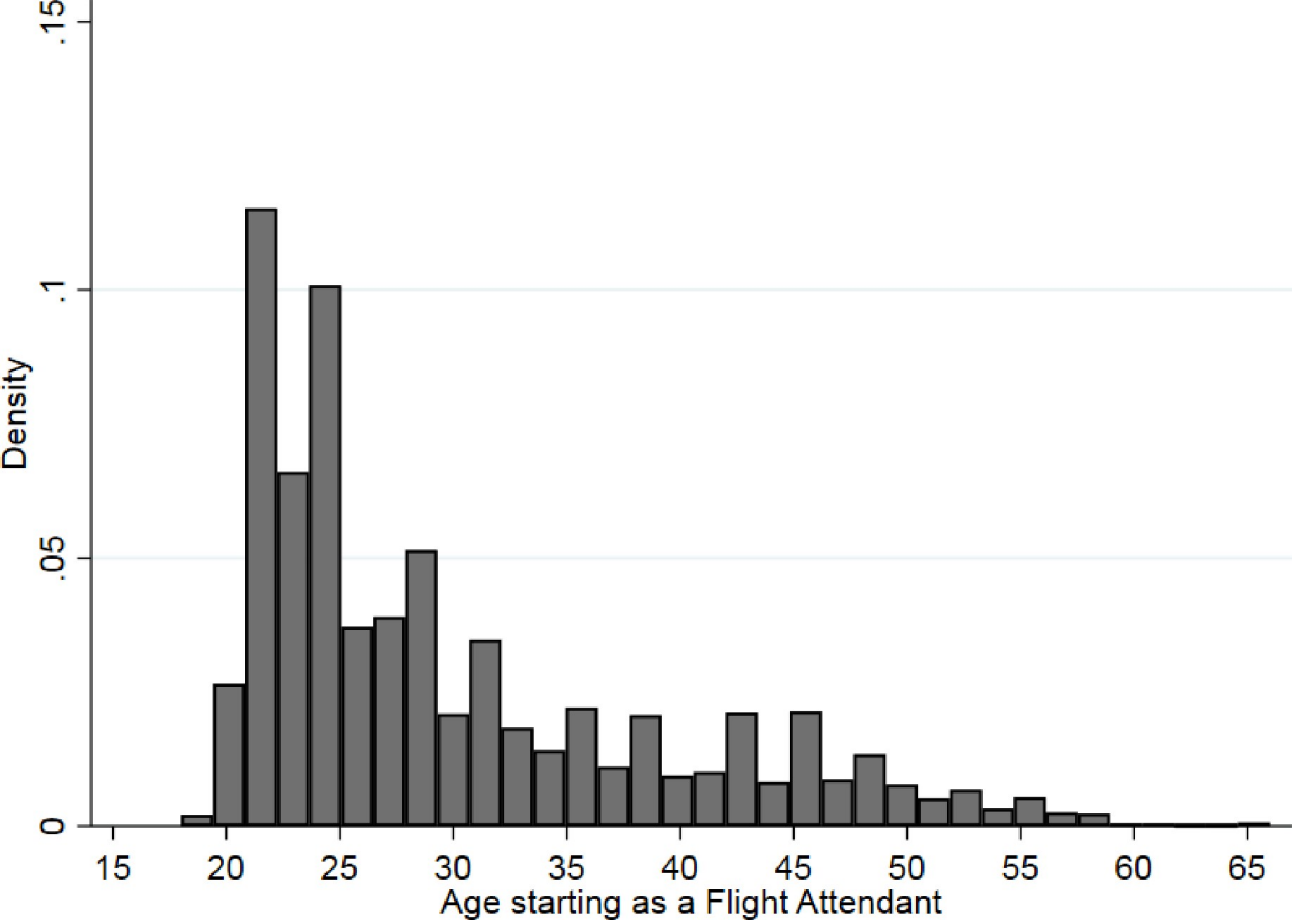
Distribution of age starting as a flight attendant among never smoking participants in the Harvard Flight Attendant Health Study, 2014–2015.

**Fig 2:**
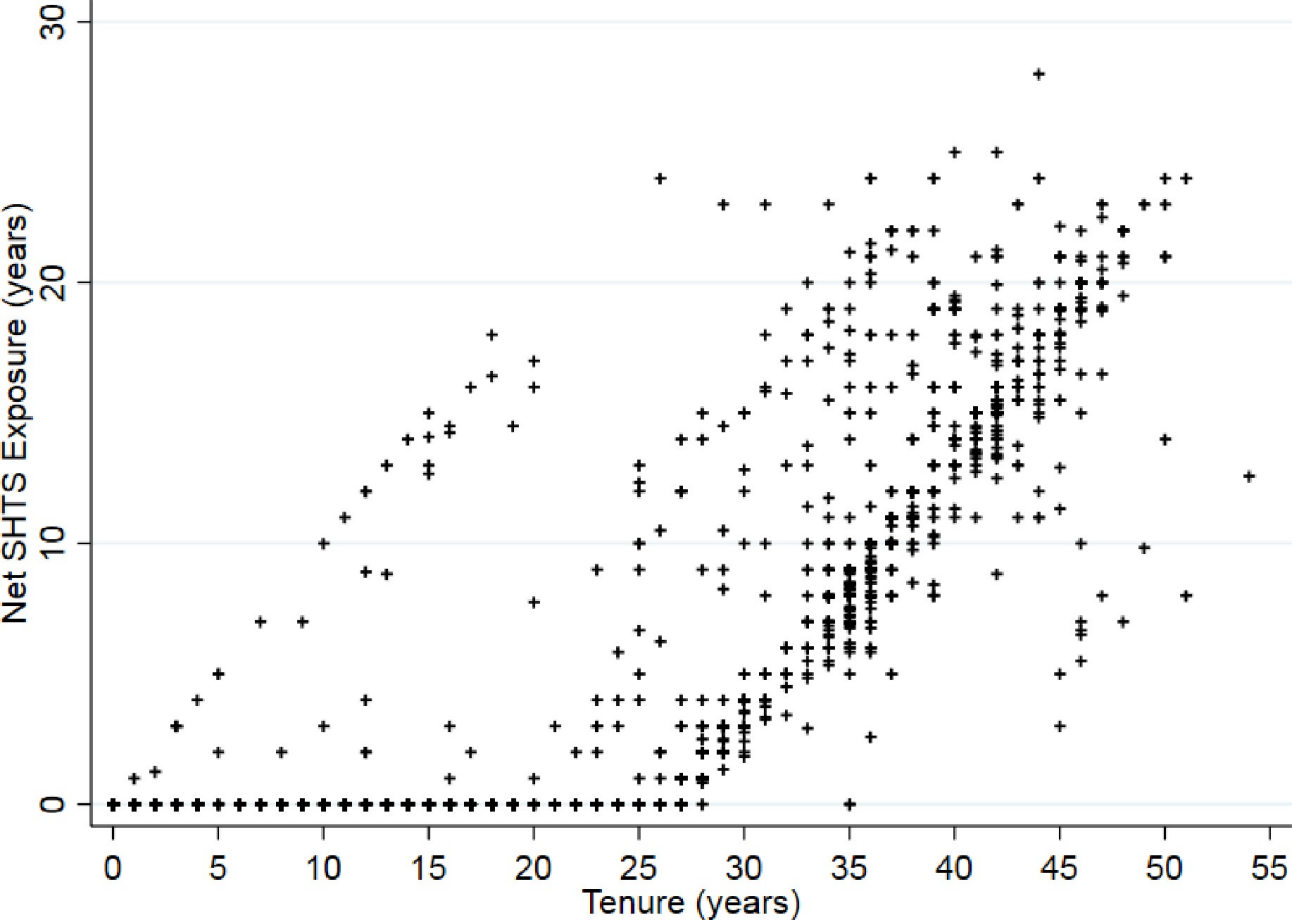
Estimated secondhand tobacco smoke exposure among never smokers in relation to years of job tenure as a flight attendant, Harvard Flight Attendant Health Study 2014–2015.

Participants most commonly started their work in their twenties, though a sizeable proportion started in their thirties as well (Fig 1). Later starting ages were less common but continued into participants’ mid to late fifties. While many participants acquired their job tenure after the start of smoking bans, tenure correlates strongly with duration of SHTS exposure (Fig 2).

**Fig 3:**
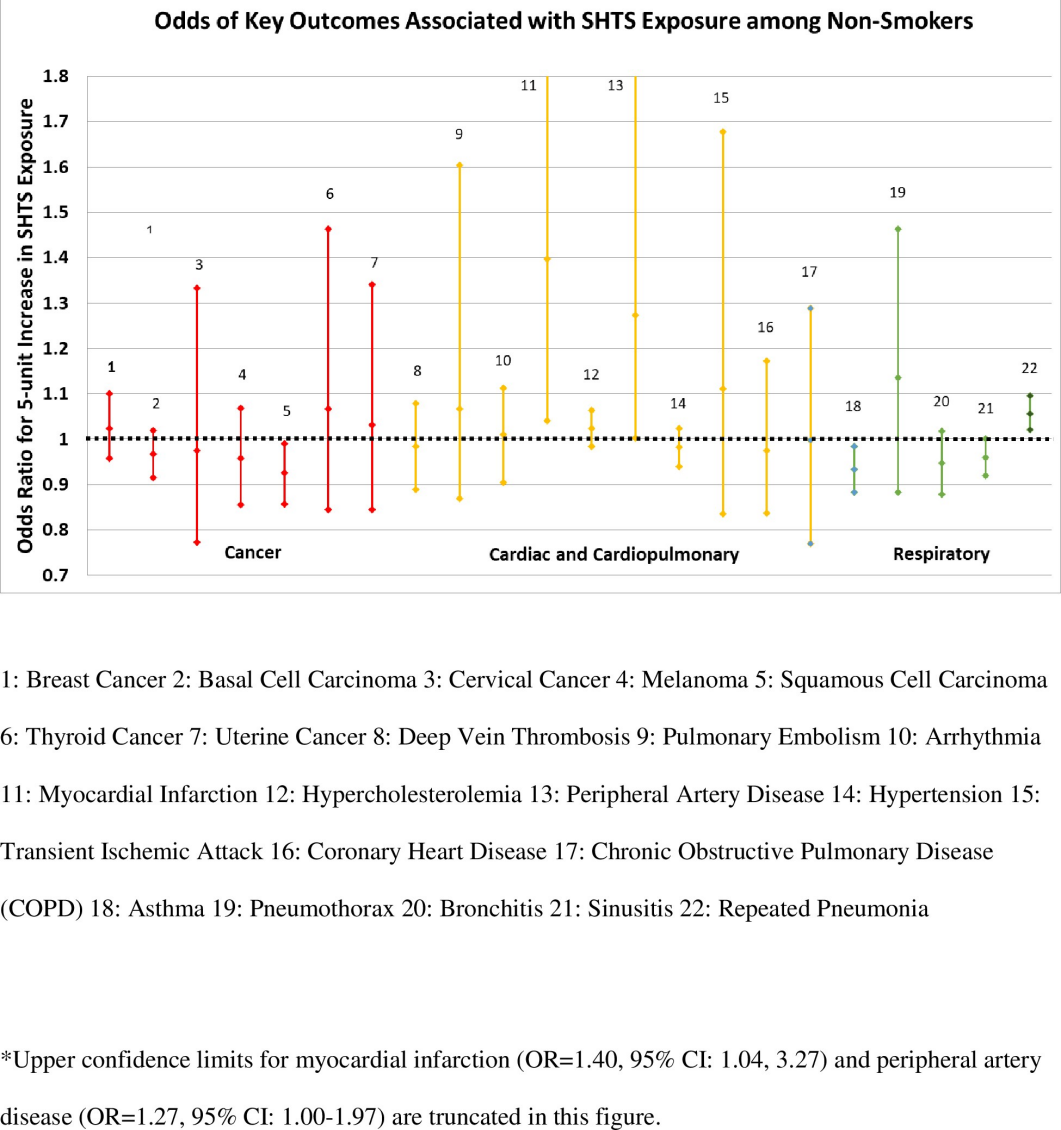
Associations between one year of secondhand tobacco exposure before 1988 and cancer, cardiac, cardiopulmonary, and respiratory health outcomes among never smoking flight attendants born before 1971 (Harvard Flight Attendant Health Study, 2014–2015).

ORs for associations between one year of SHTS exposure among never smokers and health outcomes are presented in Table 2 and Fig 3. We found no evidence of positive associations between SHTS and any cancer, and SHTS exposure was negatively related to squamous cell carcinoma (SCC; OR = 0·93, 95% CI: 0·86, 0·99). We observed associations between SHTS and cardiac outcomes, including myocardial infarction (MI; OR = 1·40, 95% CI: 1·04, 3·27), peripheral artery disease (PAD; OR = 1·27, 95% CI: 1·00, 1·97), and possibly transient ischemic attack (TIA; OR = 1·11, 95% CI: 0·84, 1·68). We found modest associations between SHTS exposure, repeated pneumonia (OR = 1·06, 95% CI: 1·02, 1·10) and possibly pneumothorax (OR = 1·14, 95% CI: 0·88, 1·46). Asthma, bronchitis, and sinusitis were not related to SHTS.

**Table 2:** Associations between SHTS exposure before 1988 and health outcomes among never smoking flight attendants born before 1971 (Harvard FAHS, 2014–2015)^1^.

Health Outcome	Exposure Odds Ratio	95% Confidence Interval	N Cases
Cancer			
Breast Cancer	1·02	0·96, 1·10	109
Cervical Cancer	0·98	0·77, 1·33	27
Uterine Cancer	1·03	0·85, 1·34	16
Basal Cell Carcinoma	0·97	0·92, 1·02	253
Squamous Cell Carcinoma	0·93	0·86, 0·99	117
Melanoma	0·96	0·86, 1·07	79
Thyroid Cancer	1·07	0·85, 1·46	20
Cardiac and Cardiopulmonary			
Deep Vein Thrombosis	0·98	0·89, 1·08	99
Pulmonary Embolism	1·07	0·87, 1·60	30
Arrhythmia	1·01	0·90, 1·11	81
Myocardial Infarction	1·40	1·04, 3·27	16
Hypercholesterolemia	1·02	0·98, 1·06	498
Peripheral Artery Disease	1·27	1·00, 1·97	16
Hypertension	0·98	0·94, 1·02	407
Transient Ischemic Attack	1·11	0·84, 1·68	13
Coronary Heart Disease	0·98	0·84, 1·17	30
COPD	1·00	0·77, 1·29	18
Lower and Upper Respiratory			
Asthma	0·93	0·88, 0·99	266
Pneumothorax	1·14	0·88, 1·46	29
Bronchitis	0·95	0·88, 1·02	148
Repeated Pneumonia	1·06	1·02, 1·10	664
Sinusitis	0·96	0·92, 1·00	475

COPD: Chronic Obstructive Pulmonary Disease; FAHS: Flight Attendant Health Study; SHTS: Secondhand Tobacco Smoke

1. Models were adjusted for three-year birth window, gender, and race, and each OR is in relation to units of a years’ employment in SHTS conditions.

Analyses using different cutoffs for SHTS exposure given staggered implementation of smoking (S2 and S3 Tables). Associations were similar when evaluating exposure before 1990. When examining exposure prior to 1995, associations were attenuated for MI (OR = 1·28, 95% CI: 1·00, 2·08) and PAD (OR = 1·13, 95% CI: 0·79, 1·97) and strengthened for TIA (OR = 1·23, 95% CI: 0·94, 2·00) and pneumothorax (OR = 1·18, 95% CI: 1·00, 1·50). We also conducted a sensitivity analysis among our full sample (S4 Table). Findings were attenuated in the sample including smokers, though associations between SHTS, MI and PAD remained elevated, with ORs of 1·14 (95% CI: 0·94, 1·50) and 1·19 (95% CI: 1·01, 1·78), respectively, and the OR for SCC remained decreased (OR = 0·93, 95% CI: 0·88, 0·99).

## Discussion

We report associations between legacy exposure to SHTS among never smoking workers and several health outcomes: MI, PAD, and repeated pneumonia. Our study is one of few to evaluate the legacy effects of workplace SHTS exposure, and to our knowledge is the most comprehensive study on this topic to date. Our findings are striking given the healthy worker effect, in which workers exhibit low morbidity because health is required to maintain employment, especially in demanding jobs [14], the low rates of obesity, hypertension and hypercholesteremia observed in our study [6,7], and the many years elapsed since implementation of smoking bans. Our results may be generalizable to other populations exposed to SHTS and, if confirmed, may inform clinical guidelines among people with such exposure histories regarding preventative health measures, screenings and health education even after many years of ceased exposure.

Flight attendants are highly understudied. In addition to their historical exposures to high levels of SHTS, cabin crew continue to be exposed to a wide range of adverse factors, including cosmic ionizing radiation at altitude, circadian rhythm disruption from shiftwork and crossing time zones, chemical contaminants, ozone, hypoxia, noise, pesticides, heavy physical and psychological job demands, and workplace harassment [15]. While we aimed to isolate the effect of SHTS through statistical methods, we also interpret our findings in light of these many exposures. For example, we found no evidence of elevated cancer risk in relation to SHTS in our study. However, as expected in the case of a rare disease with a poor five-year survival rate [16], we lacked sufficient statistical power to evaluate associations with lung cancer, the only cancer conclusively linked to SHTS [3]. Further complicating the interpretation of our results, breast and skin cancers are consistently linked to work as a flight attendant [17,18] and many cancers are related to other cabin crew exposures, including ionizing radiation and circadian rhythm disruption [19,20].

Similarly, we did not report associations between SHTS exposure and asthma, bronchitis, or sinusitis, despite some positive findings from other studies of flight crew [6,7,21]. Studies of respiratory outcomes among flight attendants exposed to SHTS have been mixed [6,7,21,22]. While SHTS is a known cause of acute respiratory symptoms, evidence is inconclusive regarding chronic respiratory or pulmonary outcomes among adults [3,23]. In addition, the risk of adverse respiratory outcomes is known to decline continually in the years after smoking cessation [24].

We observed modest associations between legacy SHTS exposure, repeated pneumonia, and pneumothorax. This is consistent with previous research that found associations between work as a flight attendant and chest infections [22], with research regarding SHTS and community-acquired pneumonia, and with the known role of active smoking in pneumonia risk [24,25]. Active smoking is also a risk factor for pneumothorax [26]. Our findings are biologically plausible, since particles in SHTS efficiently penetrate to and damage the lower respiratory airways, components of SHTS adversely impact respiratory defense mechanisms against infectious agents, smoking alters lung immunology, and studies report subclinical reduced lung function among never smoking flight attendants exposed to SHTS [3,23,27,28]. It is also possible that legacy SHTS interacts with other respiratory risk factors, such as exposure to infections from passengers [16]. Our study adds to the sparse literature on SHTS and chronic respiratory outcomes within the medical literature [23].

We report a positive association between legacy exposure to SHTS and MI, consistent with the established association between recent or current SHTS exposure and MI risk and severity [3,29]. Enactment of smoking bans in the workplace and community is related to a dramatic decrease in MI hospitalizations [30]. SHTS impacts the cardiovascular system by increasing platelet activity, reducing endothelial function, increasing arterial stiffness, promoting atherosclerosis, elevating oxidative stress and inflammation, reducing heart rate variability, and increasing the severity of tissue damage due to ischemia and infarction [3,31]. The effects of SHTS on CVD are nearly as large as those of active smoking [1]. The literature for legacy SHTS exposure is much sparser than for recent exposure in relation to CVD. Studies indicate that cardiovascular risks from active and passive smoking diminish over time but can persist for decades depending on individual factors [29,32]. This may explain why we observed associations with MI but not other cardiac outcomes. Another factor possibly underlying this discrepancy is that cabin crew have ongoing circadian rhythm disruption and noise exposure, both of which are risk factors for CVD [33,34]. This makes our findings of associations between legacy SHTS exposure and MI after many years that much more striking, especially among a sample of healthy workers with a low prevalence of MI [6,7].

The above mechanisms may also underly our observed association between legacy SHTS and PAD, consistent with reports that smoking has a greater and more persistent effect on PAD than on coronary heart disease, persisting beyond twenty years after smoking cessation [35]. Passive smoking is an important risk factor for PAD among never smokers [36]. Tenure as a flight attendant was related to the prevalence of PAD in the current wave of the FAHS, with stronger effects observed among workers with higher exposures [7].

Strengths of our study include access to a large cohort of workers with comprehensive information regarding health, work, and personal characteristics, as well as a fairly homogenous study population with regard to lifestyle and socioeconomic factors. We matched SHTS-exposed and unexposed participants according to important characteristics, including job tenure as a proxy for other occupational exposures. These factors reduce the possibility of residual confounding. Online questionnaires are an increasingly popular option in epidemiologic research, including high profile studies such as the Millennium Cohort and the Nurses’ Health Study [8]. This mode of data collection allows for validation checks, reduced data entry and coding errors, personalized question administration, convenience, security and anonymity for participants, equal or better validity compared to hard copy surveys, and collection of metadata [8].

Limitations of our study include a cross-sectional design, precluding conclusions about the direction of causality. We have included only U.S. flight attendants in our study. It is unclear to what extent our findings generalize to crew working for foreign airlines, though the timing of in-flight smoking bans is similar across many countries [2,5]. We evaluated only self-reported health outcomes due to the associated time and cost of validation through medical records. Validity of self-reported health outcomes varies by diagnosis and population characteristics [37], and misclassification of self-reported outcomes could bias results in an unpredictable direction [38]. We used tenure as a proxy for exposure. While tenure is correlated with workplace exposures such as cumulative ionizing radiation and is used as the exposure metric in many occupational studies [39], exposures are likely not uniform across participants. The generalizability of our findings to other occupations and segments of the general population is unclear, as flight attendants may differ from other groups on health-related factors. However, our study is a valuable addition to the sparse literature on the effect of legacy SHTS exposure on health, particularly in an occupational setting, and is relevant in thinking about smoking cessation efforts and preventive healthcare guidelines. An additional limitation of our study is the use of an online recruitment strategy. This means that our response rate and the representativeness of our study are unclear, though the initial FAHS cohort had a response rate of nearly 50%, and of those, approximately 40% returned for the current wave of the study.

We did not correct for multiple testing, though we employed a hypothesis-driven approach and evaluated a limited number of health outcomes. We chose to qualitatively examine the pattern of results instead. Our findings are consistent with the greater literature on this subject, which adds plausibility to the pattern of associations we observed. Larger studies with greater statistical power will be needed to confirm and expand on our initial findings.

We also did not record the date of diagnosis in our questionnaire. Hence, some conditions may have been diagnosed prior to exposure and some exposure may have occurred following a diagnosis, making the direction of the potential bias unclear, though the decades elapsed since smoking bans were implemented makes this concern much less likely. We lacked statistical power to investigate several outcomes of interest such as angina, stroke, lung fibrosis, emphysema, and several cancers. Finally, our reliance on prevalence rather than incidence of disease confuses the issues of risk and survivorship in interpreting our results.

## Conclusions

We report associations between legacy SHTS exposure going back decades and several severe cardiovascular and respiratory outcomes, including MI, PAD, and repeated pneumonia. SHTS is a preventable and modifiable exposure that continues to be a substantial cause of morbidity and premature death worldwide. Only 28 U.S. states have comprehensive public smoking bans and a large percentage of the world’s population is exposed to SHTS in the home, community, and workplace, with racial and ethnic minorities and those of lower socioeconomic status disproportionately affected [40]. This represents a tremendous public health burden in light of severe and commonly occurring health outcomes, no known safe level of exposure [3], and the fact that we observed associations despite decades elapsing since implementation of in-flight smoking bans. Our findings, if confirmed, have implications for informing smoking cessation efforts, and clarifying health education and clinical guidelines among patients with SHTS exposure histories, even in the distant past.

## Supporting information

S1 Table Demographics and baseline characteristics of ever and never smoking Harvard Flight Attendant Health Study participants born before 1971 (Wave 2: 2014–2015). (DOCX)

S2 Table Associations between SHTS exposure before 1990 and health outcomes among never smoking flight attendants (Harvard FAHS, 2014–2015). (DOCX)

S3 Table Associations between SHTS exposure before 1995 and health outcomes among never smoking flight attendants (Harvard FAHS, 2014–2015). (DOCX)

S4 Table Associations between SHTS exposure and health outcomes among never and ever smoking flight attendants (Harvard FAHS, 2014–2015). (DOCX)

S1 Text The Harvard Flight Attendant Health Study Survey. (DOCX)
