# Supplementary material for: Legacy health effects among never smokers exposed to occupational secondhand smoke
Source: PLoS One. 2019 Apr 18;14(4):e0215445. doi: 10.1371/journal.pone.0215445 (PMC6472795; doi:10.1371/journal.pone.0215445)
Supplement: S1 Table — (DOCX) [file pone.0215445.s001.docx]

S1 Table. Demographics and baseline characteristics of ever and never smoking Harvard Flight Attendant Health Study participants born before 1971 (Wave 2: 2014-2015).

|  | **Full Sample (n = 4,648)** | | **Exposed (n = 2,146)** | | **Unexposed (n = 2,502)** | |
| --- | --- | --- | --- | --- | --- | --- |
|  |  | |  | |  | |
| **Characteristic** | **N or Mean** | **% or SD** | **N or Mean** | **% or SD** | **N or Mean** | **% or SD** |
| Age (years) | 57·1 | 7·6 | 61·5 | 6·4 | 53·2 | 6·3 |
| Net Tenure (years) | 25·1 | 11·2 | 34·6 | 7·3 | 17·2 | 6·8 |
| Net Exposure (years) | 4·5 | 6·7 | 10 | 6·7 | 0 | 0 |
| BMI (kg/m^2^) | 24·3 | 4·0 | 24·3 | 4·2 | 24·4 | 3·8 |
| Sex |  |  |  |  |  |  |
| Male | 787 | 16·9 | 294 | 13·7 | 493 | 19·7 |
| Female | 3,858 | 83·1 | 1,849 | 86·3 | 2,009 | 80·3 |
| Race |  |  |  |  |  |  |
| White | 4,083 | 87·8 | 1,949 | 90·8 | 2,134 | 85·4 |
| Non-White | 565 | 12·2 | 197 | 9·2 | 368 | 14·7 |
| Current Smoker |  |  |  |  |  |  |
| Yes | 319 | 6·9 | 125 | 5·9 | 194 | 7·8 |
| No | 4,301 | 93·1 | 2,007 | 94·1 | 2,294 | 92·2 |
| Past Smoker |  |  |  |  |  |  |
| Yes | 1,576 | 34·2 | 818 | 38·5 | 758 | 30·5 |
| No | 3,034 | 65·8 | 1,309 | 61·5 | 1,725 | 69·5 |

BMI: body mass index; SD: standard deviation
