# Supplementary material for: Legacy health effects among never smokers exposed to occupational secondhand smoke
Source: PLoS One. 2019 Apr 18;14(4):e0215445. doi: 10.1371/journal.pone.0215445 (PMC6472795; doi:10.1371/journal.pone.0215445)
Supplement: S2 Table — (DOCX) [file pone.0215445.s002.docx]

S2 Table. Associations between SHTS exposure before 1990 and health outcomes among never smoking flight attendants (Harvard FAHS, 2014-2015).^1^

| **Health Outcome** | **Exposure Odds Ratio** | **95% Confidence Interval** | **N Cases** |
| --- | --- | --- | --- |
| Cancer | | | |
| Breast Cancer | 1·02 | 0·95, 1·09 | 109 |
| Cervical Cancer | 1·04 | 0·80, 1·39 | 27 |
| Uterine Cancer | 1·04 | 0·86, 1·33 | 16 |
| Basal Cell Carcinoma | 0·96 | 0·91, 1·02 | 253 |
| Squamous Cell Carcinoma | 0·92 | 0·85, 1·00 | 117 |
| Melanoma | 0·97 | 0·87, 1·07 | 79 |
| Thyroid Cancer | 1·05 | 0·84, 1·38 | 20 |
| Cardiac and Cardiopulmonary | | | |
| Deep Vein Thrombosis | 0·97 | 0·88, 1·07 | 99 |
| Pulmonary Embolism | 1·07 | 0·88, 1·52 | 30 |
| Arrhythmia | 1·02 | 0·91, 1·13 | 81 |
| Myocardial Infarction | 1·40 | 1·04, 2·67 | 16 |
| Hypercholesterolemia | 1·02 | 0·98, 1·06 | 498 |
| Peripheral Artery Disease | 1·23 | 0·93, 2·11 | 16 |
| Hypertension | 0·98 | 0·95, 1·03 | 407 |
| Transient Ischemic Attack | 1·15 | 0·87, 1·83 | 13 |
| Coronary Heart Disease | 0·98 | 0·85, 1·17 | 30 |
| COPD | 0·99 | 0·79, 1·25 | 18 |
| Lower and Upper Respiratory | | | |
| Asthma | 0·93 | 0·88, 0·99 | 266 |
| Pneumothorax | 1·16 | 0·93, 1·49 | 29 |
| Bronchitis | 0·94 | 0·88, 1·01 | 148 |
| Repeated Pneumonia | 1·05 | 1·02, 1·08 | 664 |
| Sinusitis | 0·96 | 0·92, 1·00 | 475 |

COPD: Chronic Obstructive Pulmonary Disease; FAHS: Flight Attendant Health Study; SHTS: Secondhand Tobacco Smoke

1. Models were adjusted for three-year birth window, gender, and race, and each OR is in relation to units of a years’ employment in SHTS conditions.
