# Supplementary material for: Legacy health effects among never smokers exposed to occupational secondhand smoke
Source: PLoS One. 2019 Apr 18;14(4):e0215445. doi: 10.1371/journal.pone.0215445 (PMC6472795; doi:10.1371/journal.pone.0215445)
Supplement: S4 Table — (DOCX) [file pone.0215445.s004.docx]

S4 Table. Associations between SHTS exposure and health outcomes among never and ever smoking flight attendants (Harvard FAHS, 2014-2015).^1^

| **Health Outcome** | **Exposure Odds Ratio** | **95% Confidence Interval** | **N Cases** |
| --- | --- | --- | --- |
| Cancer | | | |
| Breast Cancer | 1·03 | 0·97, 1·09 | 152 |
| Cervical Cancer | 0·99 | 0·86, 1·18 | 46 |
| Uterine Cancer | 0·95 | 0·79, 1·11 | 23 |
| Basal Cell Carcinoma | 0·97 | 0·93, 1·01 | 344 |
| Squamous Cell Carcinoma | 0·93 | 0·88, 0·99 | 155 |
| Melanoma | 0·95 | 0·86, 1·04 | 1.02 |
| Thyroid Cancer | 1·03 | 0·79, 1·38 | 25 |
| Cardiac and Cardiopulmonary | | | |
| Deep Vein Thrombosis | 0·97 | 0·91, 1·05 | 164 |
| Pulmonary Embolism | 1·10 | 0·96, 1·33 | 50 |
| Arrhythmia | 1·02 | 0·95, 1·10 | 114 |
| Myocardial Infarction | 1·14 | 0·94, 1·50 | 29 |
| Hypercholesterolemia | 1·00 | 0·97, 1·03 | 764 |
| Peripheral Artery Disease | 1·19 | 1·01, 1·78 | 25 |
| Hypertension | 0·99 | 0·96, 1·03 | 634 |
| Transient Ischemic Attack | 1·04 | 0·86, 1·28 | 26 |
| Coronary Heart Disease | 0·97 | 0·87, 1·10 | 47 |
| COPD | 1·05 | 0·93, 1·19 | 39 |
| Lower and Upper Respiratory | | | |
| Asthma | 0·94 | 0·90, 0·98 | 382 |
| Pneumothorax | 0·98 | 0·84, 1·15 | 55 |
| Bronchitis | 0·98 | 0·93, 1·04 | 214 |
| Repeated Pneumonia | 1·03 | 0·99, 1·05 | 1,028 |
| Sinusitis | 0·97 | 0·94, 1·00 | 708 |

COPD: Chronic Obstructive Pulmonary Disease; FAHS: Flight Attendant Health Study; SHTS: Secondhand Tobacco Smoke

1. Models were adjusted for age, gender, race, and current and past active smoking status, and each OR is in relation to units of a years’ employment in SHTS conditions.
