# Supplementary material for: Legacy health effects among never smokers exposed to occupational secondhand smoke
Source: PLoS One. 2019 Apr 18;14(4):e0215445. doi: 10.1371/journal.pone.0215445 (PMC6472795; doi:10.1371/journal.pone.0215445)
Supplement: S1 Text — (DOCX) [file pone.0215445.s005.docx]

FA Health V.2

First name/Given name ________________________________________________

Last name/Family name ________________________________________________

Phone number

- Example: 555-555-5555 ________________________________________________

Email address

*Don't worry.  We will not barrage you with unnecessary emails. Your email will strictly be used for the collection of your health data. We do not sell, rent, or lend our mailing list to any other group.

Please enter your email address again for validation

Home Address

- Street Address ________________________________________________
- Apt/Suite/Other ________________________________________________
- City ________________________________________________
- State/Province ________________________________________________
- Country ________________________________________________
- Zip Code/Post code ________________________________________________

What is your current domicile/hub or your last domicile/hub when you worked?

 Please enter the airport code by selecting from the boxes below.

|  |  |  |  |
| --- | --- | --- | --- |
|  |  |  |  |
| Domicile/Hub | ▼ A ... Z | ▼ A ... Z | ▼ A ... X |

What is your File number, Employee number, or bidding number?


(*We ask this so that we can avoid duplicates, ensure the highest data quality, and our integrity.)

I agree to participate in the study

- Agree
- Disagree

***Section 1: Tell us about your work***

Have you worked one flight as a flight attendant in the **past 30 days**?

- Yes
- No

Are you still a flight attendant?

- Yes, I am still a flight attendant
- No, I am no longer a flight attendant

Please choose the reason you have not worked as a flight attendant in the **last 30 days**. Please select only ONE response.

- I am only part time
- I am on involuntary furlough
- I am on voluntary furlough
- I am on sick leave
- I am out on worker's compensation disability
- I am on vacation leave or personal leave
- I am on maternity leave or paternity leave
- Other: ________________________________________________

Please choose the reason you have not worked as a flight attendant in the **last 30 days**. Please select only ONE response.

- I am working in a job other than flight attendant
- I am retired - not working any paid job or looking for work
- I am unemployed and looking for work
- I am unemployed and unable to work for health reasons
- I am unemployed and taking care of house or family
- I am going to school
- Other: ________________________________________________

What is the year that you first **began** working as a flight attendant to your best recollection? (Click on the box to select year.)

▼ 1955 ... 2015

What is the year that you **last** worked as a flight attendant on an airplane to your best recollection?

▼ 1955 ... 2015 (or to present)

Please describe your employment as a flight attendant according to major employer (airline), employment dates, and full-time status. (If taking the survey from a mobile device, please remember to scroll across.)

|  | Airline | Did you mostly work 65 hours or more per month? | | Year Started | Year Ended (or current year if still working) | If airline not listed, please tell us which airline |
| --- | --- | --- | --- | --- | --- | --- |
|  |  | Yes | No |  |  | Airline |
|  | ▼ Air Tran ... Other |  |  | ▼ 1955 ... 2015 | ▼ 1955 ... currently working |  |
|  | ▼ Air Tran ... Other |  |  | ▼ 1955 ... 2015 | ▼ 1955 ... currently working |  |
|  | ▼ Air Tran ... Other |  |  | ▼ 1955 ... 2015 | ▼ 1955 ... currently working |  |
|  | ▼ Air Tran ... Other |  |  | ▼ 1955 ... 2015 | ▼ 1955 ... currently working |  |
|  | ▼ Air Tran ... Other |  |  | ▼ 1955 ... 2015 | ▼ 1955 ... currently working |  |
|  | ▼ Air Tran ... Other |  |  | ▼ 1955 ... 2015 | ▼ 1955 ... currently working |  |

We're interested in the total amount of time you spent working as a flight attendant.  Please consider times when you took extended leaves when your employment was interrupted.

Did you ever take a leave or discontinue work as a flight attendant for an extended period of time **(at least 6 months or more)**?

|  | Yes | No | Not Applicable  (Was not a flight attendant during this time.) |
| --- | --- | --- | --- |
| Before 1988 |  |  |  |
| Between 1988 and 1998 |  |  |  |
| After 1998 |  |  |  |

You indicated that you were out for ***AT LEAST 6 months or more*** **before 1988.**Counting back in time from when you started as a flight attendant, please estimate the total months or years you were out on leave during this period (include months for any time over or under a year).
 Add up the total time of all of your leaves **before 1988.**

|  | Years | Months |
| --- | --- | --- |
|  |  |  |
| Total time out of work in years and months | ▼ 0 ... 15 | ▼ 0 ... 12 |

You indicated that you were out for ***AT LEAST 6 months or more*** **between 1988 and 1998.**Please check any year that you were out of work for at least 6 months.

|  | 1988 | 1989 | 1990 | 1991 | 1992 | 1993 | 1994 | 1995 | 1996 | 1997 | 1998 |
| --- | --- | --- | --- | --- | --- | --- | --- | --- | --- | --- | --- |
| Select any year when you were out of work **AT LEAST 6 months or more** |  |  |  |  |  |  |  |  |  |  |  |

You indicated that you were out for ***AT LEAST 6 months or more*** **after 1998.**Counting back in time from 1998 or when you started as a flight attendant (whichever is sooner), please estimate the total months or years you were out on leave during this period (include months for any time over or under a year).   Add up the total time of all of your leaves **after 1998.**

|  | Years | Months |
| --- | --- | --- |
|  |  |  |
| Sum of leave time in total | ▼ 0 ... 15 | ▼ 0 ... 12 |

Now we are interested in your recent work.  In the last year, did you regularly work **65 hours or more** per month as a flight attendant?

- Yes
- No

How many hours did you work in the last **30 days** as a flight attendant?

- 0 hours
- under 65 hours
- 65 to 74 hours
- 75 to 84 hours
- 85 to 99 hours
- 100 to 110 hours
- 111 to 120 hours
- 121 or more hours

Did you work as a flight attendant in the **last 7 days**?

- No
- Yes

Please describe your **last four days** including today (include BOTH paid and unpaid time).
 (Select one choice for each day.  If you didn't work, select "0".)
NOTE:  Since DUTY hours vary by airline, time intervals are defined below to apply uniformly among airlines.Please use the following definition of DUTY hours:
DUTY hours=time from airport check-in to 30 mins. post-flight.

|  | Approx. **DUTY** hours (begin with airport check-in to 30 mins. post-flight) | Approximately, how many duty hours were **IN FLIGHT**? | Did your trip include **INTERCONTINENTAL** travel? | | Approx., how much **SLEEP** did you get the night before? |
| --- | --- | --- | --- | --- | --- |
|  |  |  | No | Yes |  |
| Today (include only hours ALREADY worked) | ▼ 0 hours (did not work) ... 24 hours | ▼ 0 hours (did not work) ... 24 hours |  |  | ▼ 0 ... Don't Know |
| Yesterday | ▼ 0 hours (did not work) ... 24 hours | ▼ 0 hours (did not work) ... 24 hours |  |  | ▼ 0 ... Don't Know |
| Two days ago | ▼ 0 hours (did not work) ... 24 hours | ▼ 0 hours (did not work) ... 24 hours |  |  | ▼ 0 ... Don't Know |
| Three days ago | ▼ 0 hours (did not work) ... 24 hours | ▼ 0 hours (did not work) ... 24 hours |  |  | ▼ 0 ... Don't Know |

Please describe your last four days including today (include BOTH paid and unpaid time).

 (Select one choice for each day.  If you didn't work, select "0".)

|  | Did your trips include **East/West** travel with a time zone change? | | Approx., how many segments did you fly |
| --- | --- | --- | --- |
|  | No | Yes |  |
| Today (include only hours ALREADY worked) |  |  | ▼ 0 ... Don't know |
| Yesterday |  |  | ▼ 0 ... Don't know |
| Two days ago |  |  | ▼ 0 ... Don't know |
| Three days ago |  |  | ▼ 0 ... Don't know |

*In the* ***last four days*** *including today*, how many hours did you spend **IN FLIGHT TRAVELING AS A PASSENGER** for pleasure (not related to work)? (Select one choice for each day. If you didn't fly, select "0" hours.)

|  | Approximate Total Flight Hours |
| --- | --- |
|  |  |
| Today (include only hours ALREADY flown) | ▼ 0 hours ... 24 hours |
| Yesterday | ▼ 0 hours ... 24 hours |
| Two days ago | ▼ 0 hours ... 24 hours |
| Three days ago | ▼ 0 hours ... 24 hours |

Consider your work schedule in the **last 4 days (96 hours).** Did you have a layover that included a hotel stay?

- No
- Yes
- Not applicable, did not work in last 4 days

How long was the layover (that included a hotel stay) from the time the plane touched down until the time your next flight took off? (Round to the nearest hour.)

▼ less than 8 hours ... more than 24 hours

**Commuting Time**

Consider your average commute TO YOUR HUB/BASE, approximately how much time do you spend in different modes of transportation before you CHECK IN for work?   Please enter time in TOTAL to the nearest quarter hour.

|  | Hours | Minutes |
| --- | --- | --- |
|  |  |  |
| Car, Bus, Train, Shuttle, Walk, Bike, Taxi | ▼ 0 ... 12 | ▼ 0 minutes ... 45 minutes |
| Airplane | ▼ 0 ... 12 | ▼ 0 minutes ... 45 minutes |

Please describe your job as a flight attendant over the **last 12 months**.

|  | Strongly Disagree | Disagree | Agree | Strongly Agree |
| --- | --- | --- | --- | --- |
| My job requires working very fast… |  |  |  |  |
| My job requires working very hard… |  |  |  |  |
| My job requires long periods of intense concentration on the task . . . |  |  |  |  |
| My job is very hectic… |  |  |  |  |
| My work requires rapid and continuous physical activity… |  |  |  |  |
| I have enough time to get the job done… |  |  |  |  |
| I have a lot of say about what happens on my job… |  |  |  |  |
| My job requires lots of physical effort… |  |  |  |  |
| I am often required to lift very heavy loads on my job… |  |  |  |  |
| My job allows me to make a lot of decisions on my own… |  |  |  |  |
| On my job, I have very little freedom to decide how to do my work… |  |  |  |  |

Over the last 12 months, please describe whether you experienced HEALTH SYMPTOMS from the following cabin exposures? (health symptoms can include headache, difficulty thinking, trouble breathing, etc.)  

   For each item, drag the bar or click on the cell:

|  | Health Symptoms  [0=no symptoms at all] [5=moderate] [10=disabling symptoms] |
| --- | --- |

|  | 0 | 1 | 2 | 3 | 4 | 5 | 6 | 7 | 8 | 9 | 10 |
| --- | --- | --- | --- | --- | --- | --- | --- | --- | --- | --- | --- |

| Poor air quality | 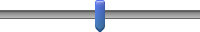 |
| --- | --- |
| Excessive noise | 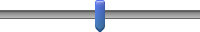 |
| Difficult passengers | 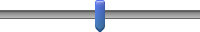 |
| Excessive heavy lifting, pulling, or pushing | 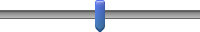 |
| Cabin pressure | 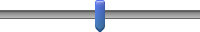 |
| Irregular work schedule | 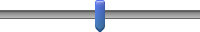 |
| Turbulence or difficult landing | 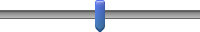 |
| Exposure to sick passengers | 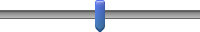 |
| Insufficient ground rest (while on a pairing or between sets of workdays) | 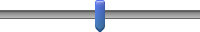 |
| Too many time zone changes | 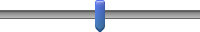 |
| Continuous sleep disruptions; no normal sleep pattern | 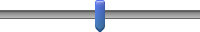 |

In the last 12 months, have you been sexually harassed at work (any type of unwelcome sexual behavior (words or actions) that creates a hostile work environment)?

- Never
- 1 time
- 2-3 times
- 4 or more times

Who did this to you? (Check any that apply.)

- A supervisor
- A pilot
- A co-worker (not a supervisor or pilot)
- An airport employee (including security screeners) or employee on a different airline
- A passenger
- Someone you know from outside work (your own partner, family, etc.)
- Others (the public, hotel staff, etc.)

In the last 12 months, have you been threatened or experienced verbal abuse at work? (e.g., yelled at, shouted at, or sworn at)

- Never
- 1 time
- 2-3 times
- 4 or more times

Who did this to you? (Check any that apply.)

- A supervisor
- A pilot
- A co-worker (not a supervisor or pilot)
- An airport employee (including security screeners) or employee on a different airline
- A passenger
- Someone you know from outside work (your own partner, family, etc.)
- Others (the public, hotel staff, etc.)

In the last 12 months, have you been sexually assaulted at work (someone used threat or force to engage in an unwanted sexual act)?

- Never
- 1 time
- 2-3 times
- 4 or more times

Who did this to you? (Check any that apply.)

- A supervisor
- A pilot
- A co-worker (not a supervisor or pilot)
- An airport employee (including security screeners) or employee on a different airline
- A passenger
- Someone you know from outside work (your own partner, family, etc.)
- Others (the public, hotel staff, etc.)

***Section 2:  Tell us about your health***

**In the last 12 months**, do YOU BELIEVE you suffered any injuries or illnesses because of your work as a flight attendant? (for example: sprains/strains, respiratory infection, ear/sinus condition, skin conditions, sleep disturbances, headaches, anxiety, depression, fatigue, other problems)

- No
- Yes
- Not Applicable

Please tell us if you experienced any of the following conditions that were related to your work as a flight attendant in the **last 12 months.** 

 (Please check all that apply for the last 12 months.)

NOTE: This is a WIDE question. 
(If taking the survey from a mobile device, please remember to scroll across.)

|  | Did you seek medical care for this injury or illness? | Did you apply for worker's compensation? | How much time did you lose from work? | Do or did you have this condition? | Did you trade flight schedules? | Did you take sick days? | Did you go on WIP (short-term or long-term disability)? |
| --- | --- | --- | --- | --- | --- | --- | --- |
|  | Yes | Yes |  | Yes | Yes | Yes | Yes |
| 1. Musculoskeletal: strain or sprain, joint aches and pains |  |  | ▼ no time lost ... 52 weeks |  |  |  |  |
| 2. Musculoskeletal: fracture, contusion |  |  | ▼ no time lost ... 52 weeks |  |  |  |  |
| 3. Respiratory: acute sinus or lung infection, common cold |  |  | ▼ no time lost ... 52 weeks |  |  |  |  |

|  | Did you seek medical care for this injury or illness? | Did you apply for worker's compensation? | How much time did you lose from work? | Do or did you have this condition? | Did you trade flight schedules? | Did you take sick days? | Did you go on WIP (short-term or long-term disability)? |
| --- | --- | --- | --- | --- | --- | --- | --- |
|  | Yes | Yes |  | Yes | Yes | Yes | Yes |
| 4. Respiratory: worsening of chronic respiratory condition (e.g. asthma, emphysema, COPD) |  |  | ▼ no time lost ... 52 weeks |  |  |  |  |
| 5. Dermatological: laceration, burns |  |  | ▼ no time lost ... 52 weeks |  |  |  |  |
| 6. Dermatological: skin rash, irritation, itching |  |  | ▼ no time lost ... 52 weeks |  |  |  |  |

|  | Did you seek medical care for this injury or illness? | Did you apply for worker's compensation? | How much time did you lose from work? | Do or did you have this condition? | Did you trade flight schedules? | Did you take sick days? | Did you go on WIP (short-term or long-term disability)? |
| --- | --- | --- | --- | --- | --- | --- | --- |
|  | Yes | Yes |  | Yes | Yes | Yes | Yes |
| 7. Ear: ear pain, ruptured drum |  |  | ▼ no time lost ... 52 weeks |  |  |  |  |
| 8. Neurological: dizziness, headaches, numbness and tingling |  |  | ▼ no time lost ... 52 weeks |  |  |  |  |
| 9. Psychological: anxiety, stress, depression |  |  | ▼ no time lost ... 52 weeks |  |  |  |  |

|  | Did you seek medical care for this injury or illness? | Did you apply for worker's compensation? | How much time did you lose from work? | Do or did you have this condition? | Did you trade flight schedules? | Did you take sick days? | Did you go on WIP (short-term or long-term disability)? |
| --- | --- | --- | --- | --- | --- | --- | --- |
|  | Yes | Yes |  | Yes | Yes | Yes | Yes |
| 10. Fatigue: sleep disturbances, tiredness |  |  | ▼ no time lost ... 52 weeks |  |  |  |  |
| 11. Cardiac: chest pain or tightness, high blood pressure |  |  | ▼ no time lost ... 52 weeks |  |  |  |  |
| 12. Vascular: deep vein thrombosis, embolism, aneurysm |  |  | ▼ no time lost ... 52 weeks |  |  |  |  |
| 13. Other: |  |  | ▼ no time lost ... 52 weeks |  |  |  |  |

Now we are going to ask you about your general health in the PAST 7 DAYS.

 In the **past 7 days**, how many days did you experience the following symptoms?

|  | Never  (0 days) | Rarely  (1-2 days) | Sometimes  (3-4 days) | Often  (5-6 days) | Everyday  (7 days) |
| --- | --- | --- | --- | --- | --- |
| *EYES, EARS, NOSE AND THROAT* Dry eyes (unrelated to contact lenses) |  |  |  |  |  |
| Itchy eyes (unrelated to contact lenses) |  |  |  |  |  |
| Eye pain (unrelated to contact lenses) |  |  |  |  |  |
| Blurred or altered vision (unrelated to glasses or contact lenses) |  |  |  |  |  |
| Sinus congestion |  |  |  |  |  |
| Sinus pain or pressure/Sinusitis |  |  |  |  |  |
| Ear pain/blockage |  |  |  |  |  |
| Ear drum rupture |  |  |  |  |  |
| Ear infection |  |  |  |  |  |
| Ringing in ears |  |  |  |  |  |
| Nosebleeds |  |  |  |  |  |
| Runny nose |  |  |  |  |  |
| Irritated/burning/sore throat |  |  |  |  |  |
| Cough |  |  |  |  |  |
| Hoarseness/voice loss |  |  |  |  |  |
| *CARDIOPULMONARY* Shortness of breath/difficulty breathing |  |  |  |  |  |
| Wheezing |  |  |  |  |  |
| Asthma attack |  |  |  |  |  |
| Bronchitis |  |  |  |  |  |
| Lung infection |  |  |  |  |  |
| Chest tightness |  |  |  |  |  |
| Chest pain |  |  |  |  |  |
| Heart racing or pounding |  |  |  |  |  |
| *GASTROINTESTINAL* Stomach pain |  |  |  |  |  |
| Nausea |  |  |  |  |  |
| Vomiting |  |  |  |  |  |
| Diarrhea |  |  |  |  |  |
| Bloating |  |  |  |  |  |
| NEUROLOGICAL/MENTAL HEALTH Fainting |  |  |  |  |  |
| Dizziness/Lightheadedness |  |  |  |  |  |
| Loss of coordination/balance |  |  |  |  |  |
| Shaking or tremors |  |  |  |  |  |
| Seizures or loss of consciousness |  |  |  |  |  |
| Numbness or tingling in the face or extremities |  |  |  |  |  |
| Severe headache |  |  |  |  |  |
| Confusion/difficulty finding words, counting, thinking |  |  |  |  |  |
| Difficulty concentrating |  |  |  |  |  |
| Loss of memory |  |  |  |  |  |
| Alterations in taste or smell |  |  |  |  |  |
| Anxiety or stress |  |  |  |  |  |
| Feeling down, depressed, or hopeless |  |  |  |  |  |
| Apathy |  |  |  |  |  |
| Irritability |  |  |  |  |  |
| Sleep disturbances, inability to stay awake or go to sleep |  |  |  |  |  |
| Unusual tiredness or fatigue |  |  |  |  |  |
| *MUSCULOSKELETAL* Calf pain/leg pain |  |  |  |  |  |
| Back pain |  |  |  |  |  |
| Hip pain |  |  |  |  |  |
| Foot pain |  |  |  |  |  |
| Shoulder pain |  |  |  |  |  |
| Elbow pain |  |  |  |  |  |
| Hand/wrist pain |  |  |  |  |  |
| Aches and pains in multiple joints |  |  |  |  |  |
| Muscle weakness |  |  |  |  |  |
| *DERMATOLOGICAL/SKIN*   Skin rashes, hives, eruptions |  |  |  |  |  |
| Itchy, irritated skin |  |  |  |  |  |
| *OTHER*   Multiple chemical sensitivity |  |  |  |  |  |
| OTHER: |  |  |  |  |  |

| Page Break |  |
| --- | --- |

Let's get more specific.  Now that you told us about your symptoms in the past week, please tell us about specific symptoms that motivated you to visit a health care provider in the LAST 12 MONTHS.

In the **last 12 months**, have you sought treatment for any of the following symptoms?   
 
Leave blank for “no”, mark for "yes" 
 

NOTE: This is a WIDE question. 
(If taking the survey from a mobile device, please remember to scroll across.)

|  | Are you currently being treated? | Do you believe these symptoms were CAUSED by your work as a flight attendant? | Is (or was) this condition AGGRAVATED by your work as a flight attendant? (YES) | Sought care in the past 12 months? |
| --- | --- | --- | --- | --- |
|  | CURRENT CARE | CAUSED | AGGRAVATED | SOUGHT CARE 12 MOS |
| *EYES, EARS, NOSE AND THROAT* Dry eyes (unrelated to contact lenses) |  |  |  |  |
| Itchy eyes (unrelated to contact lenses) |  |  |  |  |
| Eye pain (unrelated to contact lenses) |  |  |  |  |
| Blurred or altered vision (unrelated to glasses or contact lenses) |  |  |  |  |
| Sinus congestion |  |  |  |  |
| Sinus pain or pressure/Sinusitis |  |  |  |  |
| Ear pain/blockage |  |  |  |  |
| Ear drum rupture |  |  |  |  |
| Ear infection |  |  |  |  |
| Ringing in ears |  |  |  |  |
| Nosebleeds |  |  |  |  |
| Runny nose |  |  |  |  |
| Irritated/burning/sore throat |  |  |  |  |
| Cough |  |  |  |  |
| Hoarseness/voice loss |  |  |  |  |
| *CARDIOPULMONARY* Shortness of breath/difficulty breathing |  |  |  |  |
| Wheezing |  |  |  |  |
| Asthma attack |  |  |  |  |
| Bronchitis |  |  |  |  |
| Lung infection |  |  |  |  |
| Chest tightness |  |  |  |  |
| Chest pain |  |  |  |  |
| Heart racing or pounding |  |  |  |  |
| *GASTROINTESTINAL* Stomach pain |  |  |  |  |
| Nausea |  |  |  |  |
| Vomiting |  |  |  |  |
| Diarrhea |  |  |  |  |
| Bloating |  |  |  |  |
| NEUROLOGICAL/MENTAL HEALTH Fainting |  |  |  |  |
| Dizziness/Lightheadedness |  |  |  |  |
| Loss of coordination/balance |  |  |  |  |
| Shaking or tremors |  |  |  |  |
| Seizures or loss of consciousness |  |  |  |  |
| Numbness or tingling in the face or extremities |  |  |  |  |
| Severe headache |  |  |  |  |
| Confusion/difficulty finding words, counting, thinking |  |  |  |  |
| Difficulty concentrating |  |  |  |  |
| Loss of memory |  |  |  |  |
| Alterations in taste or smell |  |  |  |  |
| Anxiety or stress |  |  |  |  |
| Feeling down, depressed, or hopeless |  |  |  |  |
| Apathy |  |  |  |  |
| Irritability |  |  |  |  |
| Sleep disturbances, inability to stay awake or go to sleep |  |  |  |  |
| Unusual tiredness or fatigue |  |  |  |  |
| *MUSCULOSKELETAL* Calf pain/leg pain |  |  |  |  |
| Back pain |  |  |  |  |
| Hip pain |  |  |  |  |
| Foot pain |  |  |  |  |
| Shoulder pain |  |  |  |  |
| Elbow pain |  |  |  |  |
| Hand/wrist pain |  |  |  |  |
| Aches and pains in multiple joints |  |  |  |  |
| Muscle weakness |  |  |  |  |
| *DERMATOLOGICAL/SKIN*  Skin rashes, hives, eruptions |  |  |  |  |
| Itchy, irritated skin |  |  |  |  |
| *OTHER*   Multiple chemical sensitivity |  |  |  |  |
| OTHER: |  |  |  |  |

Now we are going to ask about your medical history.

Have you **EVER** been told by a doctor or other health care provider that you have any of the following?    
NOTE: This is a WIDE question. 
(If taking the survey from a mobile device, please remember to scroll across.)

|  | Please check box for Yes | Approximate First Year of Diagnosis | Is (or was) this condition AGGRAVATED by your work as a flight attendant? | Do you believe this condition was CAUSED by our work as a flight attendant? |
| --- | --- | --- | --- | --- |
|  | DIAGNOSED |  | AGGRAVATED | CAUSED |
| *CARDIOVASCULAR CONDITIONS*   Coronary Heart Disease |  | ▼ 1930 ... 2015 |  |  |
| Congestive Heart Failure |  | ▼ 1930 ... 2015 |  |  |
| Heart attack/Cardiac Arrest/ Myocardial Infarction (MI) |  | ▼ 1930 ... 2015 |  |  |
| Congenital Heart disease |  | ▼ 1930 ... 2015 |  |  |
| Valvular heart disease |  | ▼ 1930 ... 2015 |  |  |
| Angina pectoris |  | ▼ 1930 ... 2015 |  |  |
| Arrhythmia |  | ▼ 1930 ... 2015 |  |  |
| Cardiomyopathy |  | ▼ 1930 ... 2015 |  |  |
| Pericarditis |  | ▼ 1930 ... 2015 |  |  |
| Peripheral artery disease or claudication of legs (not varicose veins) |  | ▼ 1930 ... 2015 |  |  |
| High blood pressure (hypertension) |  | ▼ 1930 ... 2015 |  |  |
| High Cholesterol |  | ▼ 1930 ... 2015 |  |  |
| Varicose veins |  | ▼ 1930 ... 2015 |  |  |
| Stroke/ cerebrovascular accident (CVA) |  | ▼ 1930 ... 2015 |  |  |
| Transient ischemic attack (TIA) |  | ▼ 1930 ... 2015 |  |  |
| Aneurysm |  | ▼ 1930 ... 2015 |  |  |
| *DIABETES, METABOLIC AND ENDOCRINE CONDITIONS* Diabetes Mellitus |  | ▼ 1930 ... 2015 |  |  |
| Metabolic syndrome |  | ▼ 1930 ... 2015 |  |  |
| Hypothyroidism (e.g., Hashimoto's |  | ▼ 1930 ... 2015 |  |  |
| Hyperthyroidism (e.g., Graves' Disease) |  | ▼ 1930 ... 2015 |  |  |
| Thyroid nodular abnormalities (e.g., Goiter) |  | ▼ 1930 ... 2015 |  |  |
| Thyroid surgery (e.g., partial or complete thyroidectomy) |  | ▼ 1930 ... 2015 |  |  |
| *RESPIRATORY* Chronic Obstructive Pulmonary Disease (COPD) |  | ▼ 1930 ... 2015 |  |  |
| Lung fibrosis |  | ▼ 1930 ... 2015 |  |  |
| Tuberculosis |  | ▼ 1930 ... 2015 |  |  |
| Emphysema |  | ▼ 1930 ... 2015 |  |  |
| Chronic bronchitis |  | ▼ 1930 ... 2015 |  |  |
| Asthma |  | ▼ 1930 ... 2015 |  |  |
| *CANCER AND BENIGN TUMORS* Lung Cancer |  | ▼ 1930 ... 2015 |  |  |
| Oral (Mouth/Tongue/Lip) cancer |  | ▼ 1930 ... 2015 |  |  |
| Esophageal Cancer |  | ▼ 1930 ... 2015 |  |  |
| Laryngeal/Throat Cancer |  | ▼ 1930 ... 2015 |  |  |
| Breast Cancer |  | ▼ 1930 ... 2015 |  |  |
| Cancer of the uterus (endometrium) |  | ▼ 1930 ... 2015 |  |  |
| Cancer of the ovary |  | ▼ 1930 ... 2015 |  |  |
| Cancer of the cervix |  | ▼ 1930 ... 2015 |  |  |
| Prostate cancer |  | ▼ 1930 ... 2015 |  |  |
| Testicular cancer |  | ▼ 1930 ... 2015 |  |  |
| Cancer of the colon or rectum |  | ▼ 1930 ... 2015 |  |  |
| Bladder Cancer |  | ▼ 1930 ... 2015 |  |  |
| Melanoma |  | ▼ 1930 ... 2015 |  |  |
| Basal cell skin cancer |  | ▼ 1930 ... 2015 |  |  |
| Squamous cell skin cancer |  | ▼ 1930 ... 2015 |  |  |
| Leukemia (all types) |  | ▼ 1930 ... 2015 |  |  |
| Chronic Lymphocytic Leukemia |  | ▼ 1930 ... 2015 |  |  |
| Lymphoma/Hodgkin’s Disease |  | ▼ 1930 ... 2015 |  |  |
| Non-Hodgkin’s lymphoma |  | ▼ 1930 ... 2015 |  |  |
| Liver cancer |  | ▼ 1930 ... 2015 |  |  |
| Kidney cancer |  | ▼ 1930 ... 2015 |  |  |
| Stomach cancer |  | ▼ 1930 ... 2015 |  |  |
| Cancer of the pancreas |  | ▼ 1930 ... 2015 |  |  |
| Thyroid cancer |  | ▼ 1930 ... 2015 |  |  |
| Brain cancer |  | ▼ 1930 ... 2015 |  |  |
| *NERVOUS SYSTEM AND MENTAL HEALTH* Multiple Sclerosis (MS) |  | ▼ 1930 ... 2015 |  |  |
| Cognitive impairment |  | ▼ 1930 ... 2015 |  |  |
| Parkinson’s Disease |  | ▼ 1930 ... 2015 |  |  |
| Epilepsy/seizure disorder |  | ▼ 1930 ... 2015 |  |  |
| Migraine headache(s) |  | ▼ 1930 ... 2015 |  |  |
| Chronic fatigue syndrome |  | ▼ 1930 ... 2015 |  |  |
| Sleep apnea |  | ▼ 1930 ... 2015 |  |  |
| Sleep disturbances (e.g. trouble getting to sleep or staying asleep) |  | ▼ 1930 ... 2015 |  |  |
| Multiple chemical sensitivity disorder |  | ▼ 1930 ... 2015 |  |  |
| Depression |  | ▼ 1930 ... 2015 |  |  |
| Bipolar |  | ▼ 1930 ... 2015 |  |  |
| Anxiety |  | ▼ 1930 ... 2015 |  |  |
| Drug abuse |  | ▼ 1930 ... 2015 |  |  |
| Alcohol abuse |  | ▼ 1930 ... 2015 |  |  |
| Eating disorder |  | ▼ 1930 ... 2015 |  |  |
| *GYNECOLOGICAL CONDITIONS* Infertility/Trouble conceiving after 12 months |  | ▼ 1930 ... 2015 |  |  |
| Hysterectomy |  | ▼ 1930 ... 2015 |  |  |
| Polycystic Ovary Syndrome (PCOS) |  | ▼ 1930 ... 2015 |  |  |
| Endometriosis |  | ▼ 1930 ... 2015 |  |  |
| Uterine fibroids |  | ▼ 1930 ... 2015 |  |  |
| Miscarriage |  | ▼ 1930 ... 2015 |  |  |
| Congenital fetal abnormalities |  | ▼ 1930 ... 2015 |  |  |
| Premature birth of child |  | ▼ 1930 ... 2015 |  |  |
| Neo-natal death |  | ▼ 1930 ... 2015 |  |  |
| MUSCULOSKELETAL DISORDERS: ARTHRITIS, JOINT, AND AUTOIMMUNE CONDITIONS Chronic back pain |  | ▼ 1930 ... 2015 |  |  |
| Fibromyalgia |  | ▼ 1930 ... 2015 |  |  |
| Rotator cuff tear/repair |  | ▼ 1930 ... 2015 |  |  |
| Carpel tunnel syndrome |  | ▼ 1930 ... 2015 |  |  |
| Hammer toes |  | ▼ 1930 ... 2015 |  |  |
| Bunions (feet) |  | ▼ 1930 ... 2015 |  |  |
| Plantar faciitis |  | ▼ 1930 ... 2015 |  |  |
| Rheumatoid arthritis |  | ▼ 1930 ... 2015 |  |  |
| Hip fracture |  | ▼ 1930 ... 2015 |  |  |
| Osteoarthritis |  | ▼ 1930 ... 2015 |  |  |
| Osteoporosis |  | ▼ 1930 ... 2015 |  |  |
| Systemic lupus (SLE) |  | ▼ 1930 ... 2015 |  |  |
| *ORTHOPEDIC SURGERY* Hip replacement |  | ▼ 1930 ... 2015 |  |  |
| Knee replacement |  | ▼ 1930 ... 2015 |  |  |
| Knee surgery |  | ▼ 1930 ... 2015 |  |  |
| Shoulder surgery |  | ▼ 1930 ... 2015 |  |  |
| Elbow surgery |  | ▼ 1930 ... 2015 |  |  |
| Wrist surgery |  | ▼ 1930 ... 2015 |  |  |
| Back surgery |  | ▼ 1930 ... 2015 |  |  |
| Foot surgery |  | ▼ 1930 ... 2015 |  |  |
| *ALLERGIC CONDITIONS* Eczema |  | ▼ 1930 ... 2015 |  |  |
| Psoriasis |  | ▼ 1930 ... 2015 |  |  |
| Hay fever |  | ▼ 1930 ... 2015 |  |  |
| Chemical sensitivity (fragrances, detergents, etc.) |  | ▼ 1930 ... 2015 |  |  |
| General allergies (pollen, dust, mold) |  | ▼ 1930 ... 2015 |  |  |
| Food allergy |  | ▼ 1930 ... 2015 |  |  |
| GASTROINTESTINAL DISEASES   Celiac disease |  | ▼ 1930 ... 2015 |  |  |
| Ulcerative colitis/ Crohn’s |  | ▼ 1930 ... 2015 |  |  |
| Gastric or duodenal ulcer |  | ▼ 1930 ... 2015 |  |  |
| Renal/Kidney disease |  | ▼ 1930 ... 2015 |  |  |
| Kidney stones |  | ▼ 1930 ... 2015 |  |  |
| Gallbladder stones |  | ▼ 1930 ... 2015 |  |  |
| Barrett’s esophagus |  | ▼ 1930 ... 2015 |  |  |
| GERD |  | ▼ 1930 ... 2015 |  |  |
| Liver disease |  | ▼ 1930 ... 2015 |  |  |
| *EYES, EARS, NOSE AND THROAT* Chronic sinusitis |  | ▼ 1930 ... 2015 |  |  |
| Ear drum rupture |  | ▼ 1930 ... 2015 |  |  |
| Hearing loss |  | ▼ 1930 ... 2015 |  |  |
| Hearing aids |  | ▼ 1930 ... 2015 |  |  |
| Tinnitus |  | ▼ 1930 ... 2015 |  |  |
| Vertigo/Meniere's Disease |  | ▼ 1930 ... 2015 |  |  |
| Cataract(s) |  | ▼ 1930 ... 2015 |  |  |
| *OTHER CONDITIONS* Overweight/Obesity |  | ▼ 1930 ... 2015 |  |  |
| HIV/AIDS |  | ▼ 1930 ... 2015 |  |  |
| Shingles |  | ▼ 1930 ... 2015 |  |  |
| Other: |  | ▼ 1930 ... 2015 |  |  |

Has a doctor or health care provider ever diagnosed you with blood clots or deep vein thrombosis (DVT)?

- No, never
- Yes, once
- Yes, twice
- Yes, three or more times

Has a doctor or health care provider ever diagnosed you with a pulmonary embolus?

- No, never
- Yes, once
- Yes, twice
- Yes, three or more times

Has a doctor or health care provider ever diagnosed you with pneumonia?

- No, never
- Yes, once
- Yes, twice
- Yes, three or more times

Has a doctor or health care provider ever diagnosed you with pneumothorax or a collapsed lung?

- No, never
- Yes, once
- Yes, twice
- Yes, three or more times

***Section 3:  Tell us about your overall quality of life***

Would you say that in general your health is:

- Excellent
- Very good
- Good
- Fair
- Poor

Now thinking about your physical health, which includes physical illness and injury, for how many days during the **past 30 days** was your physical health not good?

▼ 0 ... 30

Now thinking about your mental health, which includes stress, depression, and problems with emotions, for how many days during the **past 30 days** was your mental health not good?

▼ 0 ... 30

During the **past 30 days**, for about how many days did PAIN make it hard for you to do your usual activities, such as self-care, work, or recreation?

▼ 0 ... 30

During the **past 30 days**, for about how many days did poor physical or mental health keep you from doing your usual activities, such as self-care, work, or recreation?

▼ 0 ... 30

During the **past 30 days**, please rate the following

|  | I never cough | I cough all the time |
| --- | --- | --- |

|  | 0 | 1 | 2 | 3 | 4 | 5 |
| --- | --- | --- | --- | --- | --- | --- |

| Coughing | 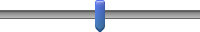 |
| --- | --- |

During the **past 30 days**, please rate the following

|  | I have no phlegm (mucus) in my chest at all | My chest is full of phlegm (mucus) |
| --- | --- | --- |

|  | 0 | 1 | 2 | 3 | 4 | 5 |
| --- | --- | --- | --- | --- | --- | --- |

| Phlegm (mucus) | 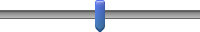 |
| --- | --- |

During the **past 30 days**, please rate the following

|  | My chest does not feel tight at all | My chest feels very tight |
| --- | --- | --- |

|  | 0 | 1 | 2 | 3 | 4 | 5 |
| --- | --- | --- | --- | --- | --- | --- |

| Chest tightness | 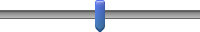 |
| --- | --- |

During the **past 30 days**, please rate the following

|  | When I walk up a hill or one flight of stairs I am not breathless | When I walk up a hill or one flight of stairs I am very breathless |
| --- | --- | --- |

|  | 0 | 1 | 2 | 3 | 4 | 5 |
| --- | --- | --- | --- | --- | --- | --- |

| Breathing | 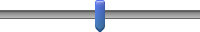 |
| --- | --- |

During the **past 30 days**, please rate the following

|  | I am not limited doing any activities at home | I am very limited doing activities at home |
| --- | --- | --- |

|  | 0 | 1 | 2 | 3 | 4 | 5 |
| --- | --- | --- | --- | --- | --- | --- |

| Activity limitation | 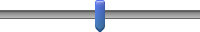 |
| --- | --- |

During the **past 30 days**, please rate the following

|  | I am confident leaving my home despite my lung condition | I am not at all confident leaving my home because of my health condition |
| --- | --- | --- |

|  | 0 | 1 | 2 | 3 | 4 | 5 |
| --- | --- | --- | --- | --- | --- | --- |

| Leaving Home | 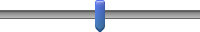 |
| --- | --- |

During the **past 30 days**, please rate the following

|  | I sleep soundly | I don't sleep soundly because of my health condition |
| --- | --- | --- |

|  | 0 | 1 | 2 | 3 | 4 | 5 |
| --- | --- | --- | --- | --- | --- | --- |

| Sleep | 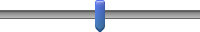 |
| --- | --- |

During the **past 30 days**, please rate the following

|  | I have lots of energy | I have no energy at all |
| --- | --- | --- |

|  | 0 | 1 | 2 | 3 | 4 | 5 |
| --- | --- | --- | --- | --- | --- | --- |

| Energy | 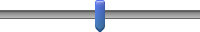 |
| --- | --- |

Are you LIMITED in any way in any activities because of any impairment or health problem?

- Yes
- No

Because of any impairment or health problem, do you need the help of other persons in handling your ROUTINE needs, such as everyday household chores, doing necessary business, shopping, or getting around for other purposes?

- Yes
- No

Because of any impairment or health problem, do you need the help of other persons with your PERSONAL CARE needs, such as eating, bathing, dressing, or getting around the house?

- Yes
- No

For HOW LONG have your activities been limited because of your major impairment or health problem?

▼ less than 1 year ... more than 20 years

What is the MAJOR impairment or health problem that limits your activities?

- a. Arthritis/rheumatism
- b. Back or neck problem
- c. Fractures, bone/joint injury
- d. Walking problem
- e. Lung/breathing problem
- f. Hearing problem
- g. Eye/vision problem
- h. Heart problem
- i. Stroke problem
- j. Hypertension/high blood pressure
- k. Diabetes
- l. Cancer
- m. Depression/anxiety/emotional problem
- n. Other impairment/problem

Over the **last 2 weeks**, how often have you been bothered by the following problems:

|  | Not at all | Several days | More than half the days | Nearly everyday |
| --- | --- | --- | --- | --- |
| 1) Little interest or pleasure in doing things? |  |  |  |  |
| 2) Feeling down, depressed, or hopeless? |  |  |  |  |
| 3) Trouble falling or staying asleep, or sleeping too much? |  |  |  |  |
| 4) Feeling tired or having little energy? |  |  |  |  |
| 5) Poor appetite or overeating? |  |  |  |  |
| 6) Feeling bad about yourself – or that you are a failure or have let yourself or your family down? |  |  |  |  |
| 7) Trouble concentrating on things, such as reading the newspaper or watching TV? |  |  |  |  |
| 8) Moving or speaking so slowly that other people could have noticed? Or the opposite – being so fidgety or restless that you have been moving around a lot more than usual? |  |  |  |  |
| 9) Thoughts that you would be better off dead or of hurting yourself in some way? |  |  |  |  |

How difficult have these problems made it for you to do your work, take care of things at home, or get along 
with people?

- Not at all
- Somewhat difficult
- Very difficult
- Extremely difficult

In the **last 30 days**, what is your average total number of hours of CONTINUOUS sleep over a ***24-hour period in a single sleep episode***?

- < 5
- 5
- 6
- 7
- 8
- 9
- 10 +
- Too difficult to estimate

In the **last 30 days**, what is your average total number of hours of CUMULATIVE sleep over a ***24-hour period? (Count all hours of sleep whether in a single sleep episode or in fragmented blocks.)***

- < 5
- 5
- 6
- 7
- 8
- 9
- 10 +
- Too difficult to estimate

During the **past month**, how often have you taken medicine  (prescribed or “over the counter”) to help you sleep?

- Not during the past month
- Less than once a week
- Once or twice a week
- Three or more times a week

Have you had a hearing test in the **past 12 months**?

- Yes
- No

How would you rate your hearing?

- Very Poor
- Poor
- Fair
- Good
- Very Good

***Section 4:  Tell us about your reproductive health***

Do you **currently** take hormones for any of the following conditions?

|  | Hormone Use | | |
| --- | --- | --- | --- |
|  | Yes | No | Not Applicable |
| Thyroid condition |  |  |  |
| Contraception |  |  |  |
| Irregular periods |  |  |  |
| Infertility |  |  |  |
| Hot flashes or other menopausal symptoms |  |  |  |
| Sex drive |  |  |  |
| Diabetes |  |  |  |
| Other: |  |  |  |

What is your sex?

- Female
- Male
- Transgender

Please indicate your assigned sex at birth.

- Female
- Male

Have you had at least one menstrual period in the **past 12 months**? (Please do not include bleedings caused by medical conditions, hormone therapy, or surgeries.)

- Yes
- No

Are you **currently** pregnant?

- Yes
- No
- Don't know

Have you **ever** tried to become pregnant for 12 consecutive months WITHOUT becoming pregnant (even if you ultimately became pregnant)?

- Yes, I was unable to become pregnant after 12 consecutive months
- No, I was able to become pregnant after 12 consecutive months
- Not Applicable because I have not tried to become pregnant for 12 consecutive months

How old were you when you first tried to become pregnant for 12 consecutive months?

(Enter age in years.)

▼ 12 ... 70

At that time did you or your partner visit a doctor to seek help getting pregnant?

- Yes
- No

Did you undergo any medical treatments or procedures to help you get pregnant? (Check any that apply)

- None
- ICSI (intracytoplasmic sperm injection)
- Intrauterine insemination
- Medication to induce ovulation, e.g. Clomiphene (e.g. Clomid), Metformin(Glucophage), Gonadotropin injections (e.g. Pergonal, Metrodin, Follstim)? (then trunks to which they took)
- In-vitro fertilization (IVF)
- GIFT or ZIFT
- Sperm donation
- Egg or embryo donation
- Other:  ________________________________________________

What is the TOTAL number of times that you have ever been pregnant? (including all miscarriages, abortions, live births and still births.)

NOT INCLUDING ANY CURRENT PREGNANCY

▼ 0 (not including any CURRENT pregnancy) ... 16

Please tell us about your pregnancies.
 
What was the outcome of your *first* 5 pregnancies?

|  | Select an outcome for each pregnancy | | | | |
| --- | --- | --- | --- | --- | --- |
|  | Pregnancy 1 | Pregnancy 2 | Pregnancy 3 | Pregnancy 4 | Pregnancy 5 |
| Single live birth |  |  |  |  |  |
| Twins |  |  |  |  |  |
| Triplets + |  |  |  |  |  |
| Miscarriage/Stillbirth |  |  |  |  |  |
| Induced abortion |  |  |  |  |  |
| Tubal or Ectopic |  |  |  |  |  |

How long did your pregnancy or pregnancies last?

|  |  | | | | |
| --- | --- | --- | --- | --- | --- |
|  | Pregnancy 1 | Pregnancy 2 | Pregnancy 3 | Pregnancy 4 | Pregnancy 5 |
| < 8 weeks |  |  |  |  |  |
| 8-11 weeks |  |  |  |  |  |
| 12-19 weeks |  |  |  |  |  |
| 20-27 weeks |  |  |  |  |  |
| 28-31 weeks |  |  |  |  |  |
| 32-36 weeks |  |  |  |  |  |
| 37-39 weeks |  |  |  |  |  |
| 40-42 (term) |  |  |  |  |  |
| 43+ weeks |  |  |  |  |  |

Have/Has your babies/baby from your first 5 pregnancies been diagnosed with any birth defect (such as a congenital anomaly, Down syndrome or TORCH syndrome)?

|  |  | | | | |
| --- | --- | --- | --- | --- | --- |
|  | Pregnancy 1 | Pregnancy 2 | Pregnancy 3 | Pregnancy 4 | Pregnancy 5 |
| Yes, diagnosed within 24 hours of birth |  |  |  |  |  |
| Yes, diagnosed more than 24 hours after birth |  |  |  |  |  |
| Maybe, still investigating |  |  |  |  |  |
| No, never diagnosed with a birth defect |  |  |  |  |  |

***Section 5:  Tell us about yourself***

What is your date of birth?

|  | Month | Day | Year |
| --- | --- | --- | --- |
|  |  |  |  |
| Date of Birth | ▼ January ... December | ▼ 1 ... 31 | ▼ 1930 ... 1997 |

Do you consider yourself to be Hispanic, Latino, or of Spanish origin?

- No
- Yes
- Don't know

What race or races do you consider yourself to be? Please select one or more.
CHECK ALL THAT APPLY.

- American Indian or Alaska Native
- Asian
- Black or African American
- Native Hawaiian or Pacific Islander
- White
- Other: ________________________________________________
- Don’t know

What is your height? (Please round to the nearest category.)

▼ 4' or 121.9 cm ... 7' or 213.4 cm

What is your weight? (Please round to the nearest category.)

▼ 75 lbs or 34.0 kg ... 350 lbs or 158.8 kg

How much schooling have you completed?

- Less than high school
- High school graduate
- Trade certificate or diploma
- GED or equivalent
- Some college but no degree
- Associate degree
- Bachelor’s degree
- Graduate school education

Are you CURRENTLY a student?

- No
- Yes, I am a part-time student
- Yes, I am a full-time student

Are you now married, widowed, divorced, separated, never married, or living with a partner?

- Married
- Widowed
- Divorced
- Separated
- Never Married
- Living with a partner, including common law

Is your partner/wife/husband employed full-time, part-time, unemployed, or retired?

- Employed full-time
- Employed part-time
- Unemployed
- Retired

Is your partner/wife/husband CURRENTLY a student?

- No
- Yes, he/she is a part-time student
- Yes, he/she is a full-time student

How many children under the age of 18 are **CURRENTLY** living in your home?

- 0
- 1
- 2
- 3
- 4 or more

Do you **CURRENTLY** smoke tobacco on a daily basis, less than daily, or not at all?

- Daily
- Less than daily
- Not at all

In the **PAST**, have you smoked tobacco on a daily basis, less than daily, or not at all? (If you smoked in the past BOTH “daily” and “less than daily", select “daily”)

- Daily
- Less than daily
- Not at all

**How often** does ANYONE smoke inside your home? Would you say daily, weekly, monthly, less than monthly, or never?

- Daily
- Weekly
- Monthly
- Less than monthly
- Never

Did your mother smoke tobacco while she was pregnant?

- No
- Yes
- Don’t Know

What are the total number of years in your **lifetime** (including as a child) that you have shared your home with someone who smoked inside your home?

▼ 0 years or I never shared a home with someone who smoked inside ... 100

Thinking about the **last 12 months,** how often do you drink . . .

|  | Never, or less than once per month | 1-3 per month | 1 per week | 2-4 per week | 5-6 per week | 1 per day | 2-3 per day | 4-5 per day | 6+ per day |
| --- | --- | --- | --- | --- | --- | --- | --- | --- | --- |
| Liquor, e.g. vodka, gin, etc. (1 drink or shot) |  |  |  |  |  |  |  |  |  |
| Light Beer, e.g. Bud Light (1 glass, bottle or can) |  |  |  |  |  |  |  |  |  |
| Beer, regular (1 glass, bottle or can) |  |  |  |  |  |  |  |  |  |
| Red Wine (5 oz glass) |  |  |  |  |  |  |  |  |  |
| White wine (5 oz glass) |  |  |  |  |  |  |  |  |  |

**On average**, what is or was the pattern of your menstrual cycles (excluding the 6 months after pregnancies or when using hormonal contraceptives)? (please average)

- Very Regular (+ or - 3 days)
- Regular (within 5-7 days)
- Usually irregular
- Always irregular
- No periods/Amenorrhea
- Used hormonal contraception
- Pregnant/Breastfeeding
